# BDNF Signaling and Pain Modulation

**DOI:** 10.3390/cells14070476

**Published:** 2025-03-22

**Authors:** Mariacristina Mazzitelli, Takaki Kiritoshi, Peyton Presto, Zachary Hurtado, Nico Antenucci, Guangchen Ji, Volker Neugebauer

**Affiliations:** 1 Department of Pharmacology and Neuroscience, School of Medicine, Texas Tech University Health Sciences Center, Lubbock, TX 79430, USA; mariacristina.mazzitelli@ttuhsc.edu (M.M.); takaki.kiritoshi@ttuhsc.edu (T.K.); peyton.presto@ttuhsc.edu (P.P.); zhurtado@ttuhsc.edu (Z.H.); nico.antenucci@ttuhsc.edu (N.A.); guangchen.ji@ttuhsc.edu (G.J.); 2 Center of Excellence for Translational Neuroscience and Therapeutics, Texas Tech University Health Sciences Center, Lubbock, TX 79430, USA; 3 Garrison Institute on Aging, Texas Tech University Health Sciences Center, Lubbock, TX 79430, USA

**Keywords:** BDNF, TrkB, pain, neuroplasticity, neuroimmune signaling

## Abstract

Brain-derived neurotrophic factor (BDNF) is an important neuromodulator of nervous system functions and plays a key role in neuronal growth and survival, neurotransmission, and synaptic plasticity. The effects of BDNF are mainly mediated by the activation of tropomyosin receptor kinase B (TrkB), expressed in both the peripheral and central nervous system. BDNF has been implicated in several neuropsychiatric conditions such as schizophrenia and anxio-depressive disorders, as well as in pain states. This review summarizes the evidence for a critical role of BDNF throughout the pain system and describes contrasting findings of its pro- and anti-nociceptive effects. Different cellular sources of BDNF, its influence on neuroimmune signaling in pain conditions, and its effects in different cell types and regions are described. These and endogenous BDNF levels, downstream signaling mechanisms, route of administration, and approaches to manipulate BDNF functions could explain the bidirectional effects in pain plasticity and pain modulation. Finally, current knowledge gaps concerning BDNF signaling in pain are discussed, including sex- and pathway-specific differences.

## 1. Introduction

Since its discovery in 1982, brain-derived neurotrophic factor (BDNF) has been extensively studied for its critical involvement in the development and maintenance of the nervous system and its essential role in neuronal survival, growth, and differentiation and neuroplasticity [1,2]. Like other neutrophins, the initial BDNF precursor protein undergoes proteolytic cleavage to generate its mature form which exhibits biologically active properties [3,4]. The molecular processes governing its production and activation are tightly regulated and involve transcriptional, translational, and post-translational mechanisms [3]. BDNF exerts its influence through the activation of the high-affinity tropomyosin receptor kinase B (TrkB) and downstream signaling cascades to modulate neuronal function [5].

In clinical studies, alterations in BDNF levels in the nervous system have been linked to neurological and psychiatric disorders [6,7,8], including Alzheimer’s disease [9,10,11], while only variations in serum BDNF have been reported in substance use disorders [12,13] and osteoarthritis patients [14].

In addition to its well-established functions in neuroplasticity and cognitive processes, BDNF has emerged as a key player in the complex realm of neuropathic pain [15]. Elevated BDNF levels have been observed in various pain conditions, contributing to increased neuronal excitability and synaptic plasticity in pain-processing circuits [16].

The objective of this review is to provide a unifying picture of BDNF’s effects in pain modulation and to identify knowledge gaps and research directions. After reviewing the source and signaling mechanisms of BDNF, information about its role in peripheral and spinal nociception and in supraspinal pain processing will be presented. The role of BDNF in related diseases will also be discussed. This review should provide a scientifically grounded perspective on BDNF as a potential target for therapeutic interventions in chronic pain and related neurological and psychiatric disorders.

## 2. BDNF Signaling

### 2.1. BDNF Synthesis, Source, and Release

The BDNF gene, located on human chromosome 11, undergoes complex transcriptional regulation [17]. The transcription of BDNF is influenced by various transcription factors, with cyclic AMP (cAMP) response element-binding protein (CREB) being a key player [18]. BNDF gene expression is neuronal activity-dependent. The BDNF gene contains a unique structure with several 5′ non-coding exons, also known as 5′untranslated regions (5′-UTRs), and one 3′ exon coding for the pre-pro-peptide [19]. The presence of multiple promoters adds an additional layer of complexity, allowing for tissue-specific and activity-dependent regulation of BDNF synthesis, as well as cellular and cognitive functions [19,20]. In rodents, there are nine functional promoters (I to IX) upstream of the nine non-coding exons, generating nine different mRNA transcripts [19]. In humans, there are also nine functional promoters but eleven exons [21]. Beyond transcriptional control, post-transcriptional processes contribute to the regulation of BDNF synthesis. RNA splicing variants give rise to different BDNF isoforms, each with distinct functional properties. Epigenetic modifications, including DNA methylation and histone acetylation, play a crucial role in shaping the chromatin landscape and, consequently, BDNF expression patterns [22]. BDNF synthesis begins with the formation of a pre-pro-peptide, pre-pro-BDNF, in the endoplasmic reticulum. Cleavage of the pre-domain results in the formation of pro-BDNF that will be transported to the Golgi apparatus for sorting and further processing in the trans-Golgi network [23,24]. Through post-translational processing, pro-BDNF is converted into the BDNF protein, which is cleaved into the mature BDNF (or BDNF) at 14 kDa [24]. BDNF is then processed through a pathway for packaging into large secretory vesicles [25]. Neuronal depolarization triggers the Ca^2+^ dependent release of BDNF-containing vesicles into the synaptic cleft, where it can act pre- or postsynaptically (see Section 2.2). This process ensures that BDNF release is tightly coupled to synaptic events, allowing it to modulate synaptic strength and plasticity in response to neuronal activity patterns [26].

BDNF gene transcription closely correlates with activity-induced Ca^2+^ increase [27], giving further evidence to activity-dependent transcription, localization, and subsequent release of BDNF. The location of BDNF in neurons can be axonal or dendritic, depending on cell type, activity, and connectivity. The different BDNF mRNA transcripts, classified in two main categories depending on the presence of either the short or long 3′-UTR, seem to also govern the localization of BDNF in the neuron. For example, long 3′-UTR transcripts prefer dendritic processes while short 3′-UTRs are directed into the soma [20]. Dendritic localization of BDNF mRNA (summarized in [28]) was found in hippocampal and cortical cells and was associated with local (dendrites) translational processes contributing to plasticity and dendritic spinal remodeling [20], whereas axonal localization was found in other groups of neurons, such as cortical axons and mossy fibers projecting to hippocampal CA3 pyramidal cells [29]. Similarly, differences in the BDNF protein localization and isoforms were observed and showed temporal features. In early life, the pro-BDNF isoform seems to be more abundant than the mature BDNF, which is the predominant isoform in adulthood. Additionally, pro-BDNF is found throughout the hippocampus in juvenile animals, while its expression is restricted to the hippocampal mossy fibers of the dentate gyrus granule cells in adult brains [20]. Expression of BDNF is regulated by other neurotrophins, including nerve growth factor (NGF) and neurotrophin-3 (NT-3) through shared signaling pathways involving tropomyosin receptor kinase (Trk) receptors and p75 neurotrophin receptor [26].

BDNF expression in the central nervous system (CNS) glia cells is less clear and somewhat controversial. Many studies have reported that spinal [30,31], brain [32], and cultured [33,34] microglia are capable of expressing BDNF mRNA and protein. However, others have found very little BDNF or TrkB receptor expression in homeostatic or lipopolysaccharide (LPS)-activated microglia in the spinal cord or in brain regions including the somatomotor cortex and hippocampus [35]. Further groups reported that spinal microglia do not express significant levels of BDNF using transcriptomic analysis [36] or *BDNF-LacZ* reporter mice [37], and still others found that resting or ATP-activated microglia do not express BDNF transcriptionally or translationally in the mouse motor cortex [38]. This discrepancy indicates a significant knowledge gap in microglial-related BDNF signaling mechanisms throughout the neuraxis.

### 2.2. Targets and Downstream Signaling

TrkB, a prominent member of the neurotrophic tyrosine kinase receptor family, serves as a pivotal mediator in the signaling effects of BDNF. TrkB consists of an extracellular domain responsible for ligand binding, a dimeric transmembrane domain, and an intracellular domain hosting the tyrosine kinase activity. Three isoforms of TrkB have been identified: the full-length receptor glycoprotein, TrkB-FL, and two truncated forms, TrkB.T1 and TrkB-T-Shc. The latter two are obtained by alternative splicing processes and lack the tyrosine kinase domain at the C-terminus [39]. The binding of BDNF to TrkB-FL is the main mechanism of action for the neurotrophic effects of BDNF. The ligand-binding domain comprises distinct regions that interact with BDNF, fostering high-affinity and selective binding between the ligand and its receptor [26,40]. The binding of BDNF to TrkB results in conformational changes in the receptor, facilitating the formation of homodimers. This dimerization event triggers the autophosphorylation of specific tyrosine residues within the intracellular domain of TrkB. The two major autophosphorylation sites on TrkB are Tyr-515 and Tyr-816 [41]. Phosphorylation at Tyr-515 provides a docking site for proteins involved in the activation of the mitogen-activated protein kinases (MAPKs) like the extracellular signal-regulated kinase (ERK) and the phosphoinositide 3-kinase/protein kinase B (PI3K/AKT) pathway. The activation of this pathway regulates gene expression, contributing to neuronal survival and differentiation. On the other hand, phosphorylation at Tyr-816 mediates the activation of the phospholipase C-γ (PLCγ) pathway involving the engagement of type II calcium/calmodulin-dependent protein kinase (CaMKII) and resulting in the activation of CREB transcription factor, which plays an important role synaptic plasticity and neuronal functions [41]. Evidence also showed the formation of a complex between the pro-BDNF, the trafficking protein sortilin, and p75 neurotrophin receptor (p75NTR) with lower affinity for BDNF than TrkB [42]. This interaction is associated with apoptosis through the c-Jun N-terminal kinases (JNK) and p53 and caspase 3 pathways or with neuronal survival via the nuclear factor kappa B (NF-kB) signaling [43,44]. Additionally, the activation of p75NTR has been involved in the development of hippocampal long-term depression (LTD) [45].

TrkB is widely expressed in the peripheral and central nervous system, including the spinal cord, brain stem, hippocampus, cerebral cortex, and cerebellum, where it mediates the physiological effects of BDNF [43,46]. BDNF/TrkB signaling is essential for survival and growth, and transgenic mutations to the TrkB gene result in severe abnormalities in the nervous system and precocious death [47,48]. Within the cell, a pool of TrkB receptors is stored in synaptic-like vesicles. Its translocation to the membrane surface on dendritic spines and axon terminals is mediated by cAMP activity promoted by the Ca^2+^ influx induced by neuronal depolarization [49]. This process occurs rapidly and guides the sensitivity of the postsynaptic neuron to BDNF [50]. Additionally, it has been shown that TrkB receptor is expressed presynaptically in glutamatergic synapses, where it modulates the neurotransmitter release [51], and on the CA3 presynaptic axons to regulate long-term potentiation (LTP) [52] (see Section 2.3). While there is also evidence for bidirectional facilitatory and inhibitory interactions between BNDF/TrkB and endocannabinoid signaling, particularly 2-arachidonoylglycerol (2-AG), in the CNS, this remains to be determined for nociceptive processing and pain conditions [53].

### 2.3. Synaptic Plasticity

Synaptic plasticity is a lasting activity-dependent functional and/or structural change in neuronal connection strength and has been considered a critical component of learning and memory [54]. Accumulating evidence suggests an important contribution of BDNF-TrkB signaling to different forms of synaptic plasticity ranging from synaptogenesis to homeostatic plasticity [55]. Among them, LTP is one of the most studied forms of synaptic plasticity, and here, we will briefly review the contribution of BDNF to LTP.

The critical involvement of BDNF in LTP was demonstrated by earlier studies showing that the genetic deletion of BDNF impaired LTP at the CA3 Schaffer collateral-CA1 synapse in the hippocampus [56,57], and the impairment was rescued by incubation with recombinant BDNF [57] or viral expression of BDNF [58]. Because of conflicting results in the literature [52,58,59,60,61], the sites (pre- or postsynaptic) of BDNF release and activation of TrkB during LTP have been controversial, and their potentially distinct roles in LTP remain to be determined. A more recent study using region-specific deletion of BDNF or TrkB revealed a specific involvement of pre- and postsynaptic BDNF-TrkB signaling in different stages of LTP at CA3 Schaffer collateral-CA1 synapses [62]. Specifically, presynaptic BDNF contributes to the induction of LTP, while postsynaptic BDNF is required for its maintenance [62]. On the other hand, presynaptic TrkB receptors are required for LTP maintenance, while postsynaptic TrkB receptors are essential for both the induction and maintenance of LTP [62]. These findings suggest that BDNF release from presynaptic terminal induces initial potentiation, while BDNF release from postsynaptic sites prolongs this potentiation. In addition to pre- and postsynaptic neurons, microglia [63] and astrocytes [64,65] have been reported as other sources of BDNF during LTP. The source of BDNF contributing to LTP could depend on spinal or brain areas and on experimental protocols.

LTP is generally divided into early (E-LTP) and late phases (L-LTP) [66,67]. E-LTP lasts up to 2–3 h, requires modification and trafficking of proteins, and is independent of de novo protein synthesis [14,15]. In contrast, L-LTP requires gene expression and local protein synthesis, and lasts hours to days [66,67]. Although the molecular mechanisms of the role of BDNF in LTP are not fully understood, evidence suggests that BDNF can modulate glutamate receptors such as α-amino-3-hydroxy-5-methyl-4-isoxazolepropionic acid receptor (AMPAR) and N-methyl-D-aspartate receptor (NMDAR). During spike-timing-dependent LTP at the CA3 Schaffer collateral-CA1 synapse, postsynaptic release of BDNF induced the insertion of the new AMPAR-containing subunit GluA1 into postsynaptic membrane [68]. This process seems to involve protein kinase C (PKC) and CaMKII-mediated phosphorylation of newly synthesized GluA1s, followed by inositol 1,4,5-triphosphate (IP3) receptor (IP3R) and transient receptor potential canonical (TRPC)-mediated Ca^2+^ transient-dependent translocation of GluA1s to the postsynaptic membrane [69,70,71]. Importantly, BDNF has been demonstrated to induce GluA1 translation through a TRPC-CaMK kinase (CAMKK)–AKT–mammalian target of rapamycin (mTOR)-dependent pathway [70]. Additionally, BDNF has been shown to enhance interactions between AMPAR subunits GluA1 and GluA2 and their scaffolding proteins (synapse-associated protein of 97 kDa (SAP97) and glutamate receptor-interacting protein1 (GRIP1)) at synapses [72], suggesting that BDNF plays a key role in the long-term maintenance of the availability of AMPAR subunits and associated scaffolding proteins at synapses. NMDAR has also been shown to critically contribute to the action of BDNF in LTP. For example, one study reported that BDNF-dependent LTP in dentate granule cells (GCs) required the activation of NMDARs and Ca^2+^ channels [73]. Similarly to the AMPAR-mediated pathway, BDNF has been shown to upregulate NMDAR subunits NR1, NR2A, and NR2B in the plasma membrane, possibly through Ca^2+^-dependent local synthesis and phosphorylation of the subunits [73,74,75,76,77]. Specifically for L-LTP, one study found that theta burst stimulation (TBS)-induced, but not HFS (four 100 Hz trains)-induced, L-LTP at the CA3 Schaffer collateral-CA1 synapse depended on BDNF-TrkB signaling-mediated modulation of subcellular distribution and nuclear translocation of the activated MAPK through cAMP-protein kinase A (PKA) signaling [78]. Interestingly, TrkB activation was not critical for the phosphorylation of MAPK in this particular form of LTP [78], suggesting a differential regulation of LTP through TrkB-independent MAPK activation and TrkB-dependent translocation. cAMP-PKA signaling has also been implicated in another type of LTP at the hilar mossy cell (MC)–GC synapse, where cAMP-PKA signaling was found to mediate LTP downstream of postsynaptic BDNF-TrkB signaling [79]. Phospholipase Cγ (PLCγ)-mediated phosphorylation of CREB and CaMKIV have also been suggested to act as key downstream targets of TrkB during both E-LTP and L-LTP at CA3 Schaffer collateral-CA1 synapse [80].

In addition to functional signaling mechanisms in LTP, BDNF has been demonstrated to induce an enlargement of CA1 dendritic spines during a spike-timing protocol-induced LTP [81], which aligns well with a study that showed that BDNF-induced mTOR-regulated reorganization of cytoskeleton mediated by the upregulation of RhoA protein, cofilin phosphorylation, and actin polymerization at CA1 dendritic spines [82]. These reports suggest that BDNF serves as a key regulator for structural synaptic consolidation underlying LTP.

**Figure 1:**
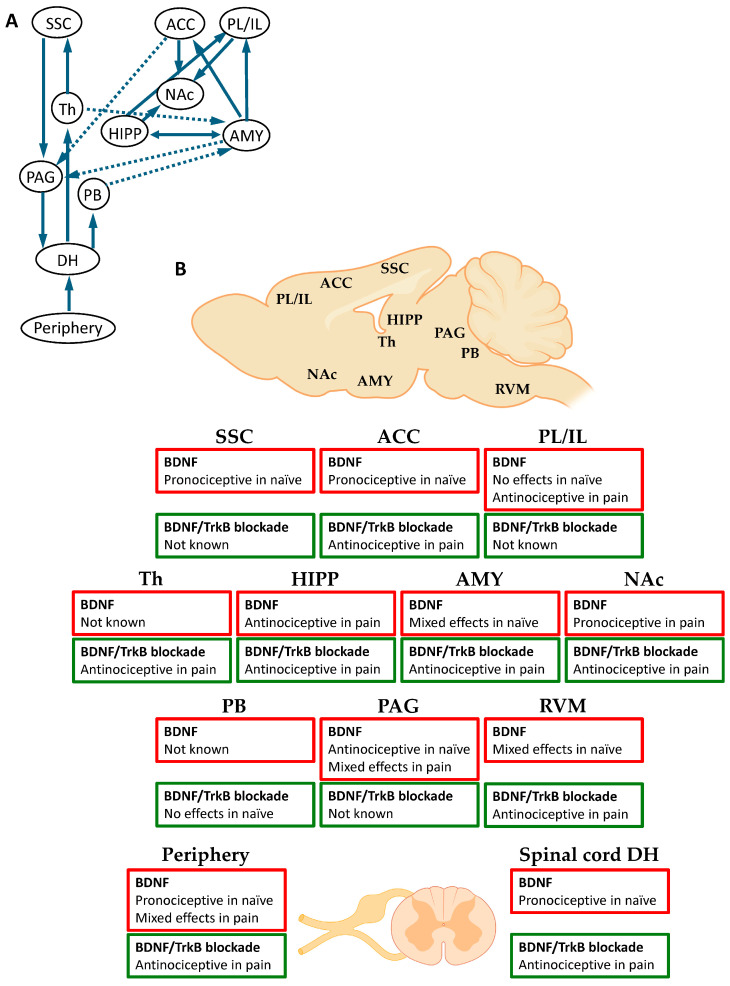
BDNF signaling in the pain system. (**A**) Elements of the pain system where BNDF signaling was explored. Dashed arrows indicate connections that were not explicitly tested. (**B**) Effects of BDNF-related manipulations tested in different brain areas, spinal cord, and periphery. Red boxes, BDNF; green boxes, BDNF/TrkB blockade. ACC, anterior cingulate cortex; AMY, amygdala; DH, dorsal horn; HIPP, hippocampus; NAc, nucleus accumbens; PAG, periaqueductal gray; PL/IL, pre/infra limbic cortex; PB, parabrachial nucleus; RVM, rostral ventromedial medulla; SSC, primary somatosensory cortex; Th, thalamus.

Although most of the evidence described above comes from studies on hippocampal LTP, important contributions of BDNF-TrkB signaling to LTP have also been reported outside of the hippocampus, including key areas for pain processing such as the spinal dorsal horn [63,83,84], nucleus accumbens (NAc) [85], medial prefrontal cortex (mPFC) [86], and anterior cingulate cortex (ACC) [87]. These findings implicate BDNF in pain-related synaptic plasticity underlying pathological pain [88]. Accumulating evidence suggests the critical contribution of the BDNF signaling to pain mechanisms at the peripheral (Section 3), spinal (Section 4), and supraspinal (Section 5) levels (Figure 1), but the role of BDNF in non-LTP plasticity (Section 2.3) is relatively unknown compared to its role in neuroimmune signaling (Section 2.4).

### 2.4. Neuroimmune Signaling

The field of pain research has overwhelmingly focused on the role of neurons in pain-related signaling mechanisms. However, a growing area of research is on the contribution of non-neuronal cells, such as astrocytes and microglia, due to their influence on and response to neuronal activity changes within nociceptive pathways [89,90]. In this sense, it is important to address the impact of BDNF in glia and other cells of relevance in pain modulation. Here, we describe the localization of BDNF in neuroimmune cell types, its mechanisms of action in these cells, and its role in neuroimmune signaling at baseline.

#### 2.4.1. Expression of BDNF on Neuroimmune Cell Types

As discussed throughout this review, BDNF is widely distributed in many neuronal cell types throughout the peripheral and central nervous systems. Under physiological conditions, BDNF binds to the TrkB and other receptors and triggers distinct downstream pathways (see Section 2.2). This complex network of signaling cascades can influence the regulation of inflammatory cytokines in neuroimmune cells, further impacting endogenous inflammatory responses.

*Peripheral Nervous System*

Within the peripheral nervous system (PNS), BDNF has been shown to be secreted by activated macrophages and Schwann cells. Macrophages are versatile cells that play a crucial role in the innate immune system, originating from precursor cells in bone marrow and migrating to sites of injury to phagocytose pathogens, modulate inflammation, and signal tissue repair. They exist in several subtypes, resting in an inactivated (M0) state or polarized into pro-inflammatory (M1) or anti-inflammatory (M2) phenotypes in response to stimuli [91,92,93]. Immunohistochemistry and flow cytometry experiments in male rats revealed very low expression of BDNF protein in inactivated M0 macrophages, whereas M1 and M2 macrophages activated by myocardial infarction showed strong BDNF mRNA and protein expression [94]. Another study found that the application of BDNF to cultured macrophages from male mice stimulated the expression of both BDNF and its TrkB receptor, suggesting that BDNF/TrkB signaling plays an important role in the activation processes of macrophages through potential autocrine mechanisms [95]. However, macrophage expression of BDNF in pain states remains underexplored.

Schwann cells are the primary glial cell type of the PNS and play a critical role in the support and maintenance of nerve functions, chiefly through their ability to form myelin sheaths around peripheral nerves to provide electrical insulation to axons and increase conduction of action potentials [96]. Schwann cells are also involved in the regeneration and repair of damaged nerves through clearing debris and releasing mediators, including BDNF [97,98,99,100]. Rat Schwann cell cultures that were exposed to the passively secreted progesterone metabolite allopregnanolone showed significantly higher levels of BDNF mRNA expression and increased protein levels of the precursor proBDNF and mature BDNF [101]. Increased expression of BDNF has also been shown to promote the proliferation of Schwann cells [102,103], suggesting that, as with macrophages, BDNF may act on Schwann cells in an autocrine fashion.

*Central Nervous System*

Microglia are the resident immune cells of the CNS and play a key role in the maintenance of neural homeostasis and in the response to injury or disease. As highly dynamic cells, they possess many critical functions such as the phagocytosis of pathogens, the regulation of synaptic pruning, and the release of various cytokines and growth factors—including BDNF—that can either support or inhibit neural cell survival and differentiation [104,105]. Therefore, microglia can serve either neuroprotective or neurotoxic mechanisms [106]. Following peripheral nerve injury, intrathecal (i.th.) injection of BDNF was found to significantly upregulate BDNF protein expression and trigger M1 polarization of spinal dorsal horn microglia [107], suggesting a role for BDNF autocrine regulatory mechanisms in the CNS just like in the PNS. Peripheral nerve injury has also been shown to upregulate BDNF mRNA expression in somatosensory cortex (S1) microglia in male mice [108]. BDNF protein was also upregulated in the ACC and S1 in male rats with inflammatory pain [109]. Increased BDNF has additionally been shown to contribute to increased spinal microglia activation in different models of pain (see Section 4.1). The data suggest that BDNF expression in the spinal cord may be associated predominately with microglia’s pro-inflammatory phenotype, though further studies are needed to characterize its expression in supraspinal regions.

Astrocytes are versatile CNS cells that play a critical role in supporting neuronal function and maintaining the brain’s microenvironment; this includes providing structural and metabolic support, maintaining ion homeostasis, recycling neurotransmitters, forming the blood–brain barrier, and modulating synaptic activity [110,111,112]. One study proposed that cortical and hippocampal astrocytes may express BDNF during development but cease during adulthood, or small subsets of astrocytes may transiently express BDNF; however, astrocytes were found to predominately express the TrkB receptor in these regions [35]. Other in vitro studies have shown that brain (hippocampal and cortical) astrocytes can express BDNF both under normal conditions [27] and following injury [113,114]. Under pain conditions, astrocytic expression of BDNF has been demonstrated in the ACC and primary sensory cortex but was not compared to normal conditions [109] (see Section 5.1). Further exploration is needed to determine BDNF expression patterns in other regions throughout the neuraxis at baseline and in pain states.

Oligodendrocytes are the primary myelinating cell type in the CNS; this role allows them to supply vital nutrients to neurons, promote long-term axonal integrity, and coordinate the timing and strength of neuronal signaling [115,116,117]. A small portion of hippocampal oligodendrocytes were found to express BDNF or the TrkB receptor [35], though BDNF expression has been reported in cortical oligodendrocytes [118,119]. Most spinal oligodendrocytes were found to produce BDNF protein under normal conditions and upregulate its expression both 1 day and 1 week following spinal cord injury [120]. However, the role of oligodendrocytes and oligodendrocytic BDNF expression in pain conditions remains to be determined.

#### 2.4.2. BDNF and Neuroimmune Signaling

*Peripheral Nervous System*

Application of BDNF was found to promote M2 polarization of mouse [121] and human [122] cultured macrophages through repression of the signal transducer and activator of transcription 3 (STAT3) pathway and inhibition of pro-inflammatory interleukin (IL)-1β, tumor necrosis factor (TNF)-α, and IL-6 expression. Furthermore, mice with diabetic coronary atherosclerosis had a downregulation of BDNF mRNA and an increased differentiation of M1 macrophages compared to control, whereas an overexpression of BDNF induced the differentiation of M2 macrophages [123]. Knockout of p75NTR significantly reduced the secretion of pro-inflammatory cytokines in both LPS-stimulated and unstimulated cultured macrophages, suggesting that p75NTR mediates the effects of BDNF in normal and inflammatory conditions [122]. In a mouse model of activity-induced muscle pain, muscle fatigue metabolites promoted macrophages to release IL-1 β, which then promoted the release of BDNF from primary dorsal root ganglion (DRG) neurons, though, interestingly, this finding was male-specific [124].

In Schwann cells, BDNF release has been shown to be dependent on P2X purinoceptor 4 (P2X4) activation. Overexpression of this receptor in male mouse Schwann cells accelerated nerve remyelination via BDNF release following a nerve crush injury [97]. Schwann cell BDNF secretion is also dependent on the T-type voltage-gated calcium channel [100]. Contrarily, the inhibition of the P2X4 prevents an increase in BDNF release [125]. The release of BDNF by Schwann cells was found to regulate PKCε in DRG neurons via TrkB activation in a paracrine manner [101], which has been shown to lead to the sensitization of primary afferent nociceptors [126]. Together, the data suggest that BDNF’s role in the PNS may be beneficial for reducing inflammation and aiding in nerve repair, though pain-related BDNF neuroimmune signaling mechanisms remain to be determined.

*Central Nervous System*

Mechanisms controlling microglial BDNF signaling are complex and have been well-reviewed by others [26,127,128]. Many stimuli, such as extracellular nucleotides [30,129,130] in spinal microglia and pro-inflammatory compounds [131] in cultured microglia, have been shown to stimulate BDNF secretion from microglia. Following peripheral nerve injury, BDNF was found to regulate spinal microglial autophagy through the AKT/mTOR pathway [108]. It has been proposed that nerve injury in male rats induces ATP release, activates microglial P2X4, and leads to spinal microglial BDNF secretion [132]. BDNF binding to the TrkB receptor on lamina I neurons may then interrupt chloride homeostasis and lead to increased intracellular chloride levels via a downregulation of the K^+^/Cl^−^ cotransporter KCC2. These alterations may ultimately disinhibit GABA- and glycine-mediated synaptic transmission [132], leading to increased excitability and eventual action potential firing in lamina I neurons, which has been shown to promote the development of neuropathic pain [105]. BDNF has also been implicated in crosstalk between microglia and astrocytes in the spinal cord. Intrathecal injection of exogenous BDNF promoted spinal microglial (and astrocytic) activation and a subsequent increase in pro-inflammatory cytokine expression in a cyclophosphamide-induced cystitis model [133]. Similarly, increased signaling between the astrocytic colony-stimulating factor-1 (CSF1) and the microglial CSF1 receptor was found 6 h after injury in a chronic post ischemic pain model; this microglial activation led to a subsequent increase in the synthesis and secretion of BDNF, which heightened neuronal activity in the spinal dorsal horn [134]. Exogenous microglial activation caused similar effects in naïve rats, whereas inhibition of this astrocyte–microglia crosstalk suppressed BDNF upregulation and neuronal activity in ischemic rats [134]. These effects have predominately been explored in male animals. It is important to note that females do not exhibit the same upregulation of microglial P2X4 as males in neuropathic pain conditions [135]; therefore, potential sexually dimorphic mechanisms may occur with regard to microglial BDNF signaling in this region.

BDNF signaling mechanisms in astrocytes and oligodendrocytes have not been well characterized. In hippocampal or perirhinal cortex slices, proBDNF has been reported to be endocytosed by astrocytes in a p75-dependent manner [65,136]. The TrkB.T1 also mediates the storage of endocytosed proBDNF in astrocytes [137]. Following intracellular cleavage of neuronal pro-BDNF by astrocytes, mature BDNF was shown to be released and act on the TrkB receptor of adjacent neurons [65]. Astrocytic BDNF release may also be induced through the inhibition of their inwardly rectifying potassium (Kir) 4.1 channels [138]. Other factors that induce astrocytic release of recycled BDNF include glutamate release from the presynaptic terminal [139,140] and ATP through the P2X7 [141]. Oligodendrocyte modulation of neurotransmitter release was shown to utilize BDNF derived from oligodendrocytes and TrkB receptor signaling at presynaptic brainstem terminals that express the vesicular glutamate transporter VGluT1 [142]. Injection of a lentiviral vector expressing BDNF into the spinal cord was found to promote M2 polarization of local macrophages in a mouse spinal cord injury model [143]. Though further studies are needed, particularly among supraspinal regions, the neuroimmune-related signaling of BDNF in the CNS seems to confer detrimental effects. Most spinal BDNF actions appear to involve glia-to-neuron signaling pathways.

## 3. Peripheral Nociception

BDNF has consistently been linked to pronociceptive processes in the peripheral nervous system, but the regulatory mechanisms of peripheral BDNF levels in pain conditions remain a substantial knowledge gap.

### 3.1. Expression and Localization

Early immunostaining analyses found that BDNF was equally expressed in small-, medium-, and large-size DRG cells in rats under normal conditions [144,145], while a subsequent study using transgenic manipulation to express β-Gal under the control of the BDNF promoter (*BDNF^LacZ/+^* mice) demonstrated that BDNF is expressed only in a specific group of small-to-medium-sized nociceptors containing other neuropeptides that play important roles in nociceptive signaling [37,146], pointing to some inconsistencies that might be due to technical issues related to the use of non-specific antibodies. Electron microscopy immunohistochemical analyses revealed that approximately 55% of BDNF-immunoreactive neurons (L4–L5 DRG) showed positive signals for calcitonin gene-related peptide (CGRP), and many primary afferents in the laminae I–II of the spinal cord co-express CGRP and the low-affinity p75NTR, suggesting a parallel release of BDNF alongside neurotransmitters from specific nociceptive primary afferents in the spinal cord [147]. Importantly, BDNF did not seem to colocalize with the non-peptidergic marker isolectin B4 (IB4) [148]. A study using *BDNF-LacZ* reporter mice found that β-Gal positive signal, a marker of BDNF-expressing neurons, predominantly colocalized with NF200, a marker of myelinated fibers, suggesting that BDNF is contained in myelinated primary afferents and its expression is limited to the subclasses of nociceptors and pruritoceptors [37]. Notably, BDNF did not colocalize with glial markers in the DRG in a double transgenic *BDNF-Cre/floxed-tdTomato* mouse model [37]. Electron microscopy showed TrkB-FL receptor expression on primary afferent fibers of both mice and rats [144,149].

Peripheral BDNF expression and release has been implicated in various pain conditions, including neuropathic, inflammatory, visceral, bone, and musculoskeletal pain [150]. In neuropathic (sciatic nerve photochemical injury, Gazelius model) rats with allodynia, BDNF was upregulated in small- and medium-sized DRG cells as compared to the non-allodynic group that showed increased BDNF signals preferentially in large-sized neurons [145]. Significant BDNF immunoreactivity upregulation was found in lumbar (L4–L5) DRGs and in the axonal terminals in the L4/L5 dorsal horn in neuropathic (chronic constriction injury, CCI; and spinal nerve ligation, SNL) [151,152,153] and NGF-induced [154] or Complete Freund’s Adjuvant (CFA) inflammatory [155] pain models, as well as in cervical DRGs in the bilateral cervical facet joint distraction pain model [156], pointing to the involvement of the endogenous BDNF in the initiation of different types of pain. BDNF and TrkB positive signals were colocalized in DRGs of diabetic rats (model established by the combination a specific diet with a systemic streptozotocin (STZ) injection) [157]. Similarly, the protein and mRNA levels of BDNF and TrkB were increased in DRGs of STZ-induced diabetic animals compared to the control group, and chronic (6 weeks) exogenous administration of BDNF or a BDNF-sequestering fusion protein (TrkB–Fc) failed to revert these effects [158]. CFA inflammatory pain or TNF-α intraplantar injection induced the upregulation of BDNF and TrkB receptor signals in rat DRGs compared to the contralateral side, while primary (L1–L6) DRG cultures chronically (24 or 48 h) treated with TNF-α showed increased the mRNA and protein levels of BDNF and TrkB receptor, as well as enhanced the release of BDNF [159]. After the induction of inflammatory pain (formalin and CFA), proBDNF protein levels were found to be upregulated in local tissue of mice, while BDNF was downregulated [160], suggesting an impairment in BDNF cleavage processes in the inflamed area.

Several studies have suggested that peripheral BDNF may be linked to different signaling pathways in pain conditions. For example, BDNF immunoreactivity or mRNA levels were associated with P2X4 and P2X7 [125,152], NGF and TrkA [153], p75NTR [161,162] and mTOR [162], and ERK1/2 and tumor necrosis factor receptor 1 (TNFR1) signals [159], while changes in BDNF total protein levels were related to Huntington-associated protein 1 (HAP1)-induced reductions in L-type calcium channel (Cav1.2) signaling [163] in pain. Finally, BDNF release by peripheral immune cells, particularly Schwann cells, was linked to TrkB-mediated PKCε signaling [101].

### 3.2. Cellular Functions

Only a few studies have addressed the peripheral neuronal actions of BDNF in pain processing. In a saphenous nerve–skin preparation obtained from normal rats, acute BDNF application induced sensitization to the heat stimulation of the C-fibers [164], suggesting its crucial role in peripheral heat responses. Moreover, BDNF significantly increased the release of CGRP and substance P in (L1–L6) DRG cultures pretreated chronically (48 h) with TNF-α compared to their untreated (non-TNF-α-treated) counterpart, although it failed to potentiate the release of the two mediators in the TNF-α group when applied for 30–60 min [159]. In contrast, chronic (6 weeks) i.th. administration of BDNF in STZ-induced diabetic rats effectively reversed the changes in acutely cultured DRG neurons induced by the neuropathic pain model, which included depolarized resting membrane potential (RMP), reduced rheobase, and enhanced action potential frequency [158], pointing to antinociceptive BDNF properties in pain conditions, which may be mediated by compensatory mechanisms induced by the prolonged treatment. These effects were not observed in the presence of TrkB–Fc in neuropathic animals or in the control (non-neuropathic) group [158]. Conditional *Bdnf* knockout mice obtained by the preferential deletion of BDNF from peripheral sensory neurons (Avil-CreERT2 mice) did not show changes in the responses of spinal L3–L5 dorsal horn wide-dynamic-range neurons evoked by mechanical or thermal stimulation under normal conditions [165]. Although little information is currently available about the action of peripheral BDNF on pain processing, these electrophysiological results point to the facilitatory effects of BDNF in the development of pain-related responses (Table 1).

### 3.3. Behavioral Studies

In the postnatal life period, BDNF plays essential functions for the survival of peptidergic and non-peptidergic nociceptors of the spinal L1 and L4 segments [166]. Mice carrying mutations of the *Bdnf* gene (homozygous mutant, *Bdnf*^−/−^) lost approximately half of all nociceptive neurons during the first 2 weeks of life, and *Bdnf*^+/−^ heterozygous animals, which were used instead of the *Bdnf*^−/−^ homozygous mutant animals because of their poor life expectancy, exhibited decreased nociceptive behavior in the hot plate test compared to their wild-type counterpart under basal conditions [166]. Conditional *Bdnf* knockout (*Avil-CreERT2* to selectively delete BDNF from primary sensory neurons) mice did not have altered baseline nociceptive responses or motor functions [37,165]; there was no difference between male and female mice, whereas the conditional *Bdnf* knockout males, but not females, showed increased thermal withdrawal latencies in the tail immersion test [37]. No significant differences were found between the knockout and wild-type groups in pruritogen-induced scratching responses or in pain-like behaviors in spared nerve injury (SNI)- or paclitaxel-induced neuropathies or the CFA inflammatory model [37]. *Avil-CreERT2* male mice showed reduced nociceptive responses in the second phase of the formalin test [37,165], while (knockout) females displayed diminished histamine-induced scratching [37], pointing to complex and sexually dimorphic BDNF functions in peripheral pain processing. Moreover, *Avil-CreERT2* male and female mice showed a reversal of mechanical hypersensitivity at the chronic stage of the SNL model of neuropathic pain, while no differences were found in the partial sciatic nerve ligation (pSNL) model [165]. In the same study, the prolonged hyperalgesia in the priming model achieved by the intraplantar injection of prostaglandin E2 (PGE2) following an intraplantar administration of carrageenan was lost in BNDF knockout mice [165], suggesting that BDNF effects on chronic pain may depend on the type of injury. When BDNF was injected into the plantar surface of the rat paw, it resulted in hyperalgesic responses compared to the vehicle-treated paw in the same animals [164] and induced mechanical allodynia in naïve mice [97]. Similarly, the application of BDNF onto intact L5 DRGs by osmotic pumps promoted a long-lasting (7 days) mechanical allodynia in uninjured rats [160], pointing to a strong contribution of BDNF to the development of peripheral pain responses. Interestingly, exogenous BDNF had no effects in the sciatic nerve crush injury mouse model [97], while blockade of BDNF signaling by the injection of an antibody against BDNF on L5 DRGs reversed mechanical allodynia in a L5 spinal nerve lesion rat model [160]. Importantly, intraplantar application of an adenovirus vector encoding proBDNF gene (Ad-proBDNF), but not (mature) BDNF, promoted nociceptive responses (licking and flitching) in combination with a low (0.5%) dose of formalin and decreased mechanical withdrawal thresholds in normal mice [167]. Pretreatment with a polyclonal anti-human proBDNF antibody (poly-Ab-proBDNF) inhibited both phases of the formalin- and visceral pain (induced by the systemic injection of acetic acid)-induced pain responses [167], suggesting a critical role of proBDNF in peripheral nociception. Intra-articular injection of BDNF into the rat knee had no effects in naïve animals but exacerbated weight-bearing impairments and mechanical allodynia in an osteoarthritis pain model induced by monoiodoacetate injection; intra-articular TrkB-Fctreatment partially reversed weight-bearing asymmetries and mechanical allodynia in surgically or monoiodoacetate induced osteoarthritis pain models [168]. Little has been studied with regard to the role of neuroimmune signaling mechanisms involving BDNF on pain-related behaviors in the periphery. Selective inhibition of BDNF signaling in macrophages via the neurotrophin inhibitor Y1036 prevented hypersensitivity induced by the application of nucleus pulposus to the sciatic nerve in male and female mice [169].

Collectively, these reports suggest that BDNF from peripheral afferents has facilitatory effects in the development of pain-related behaviors under normal conditions, although some inconsistent results were obtained with exogenous BDNF applications into the knee joint, perhaps pointing to important differences in routes of administration and tissue-specific actions with respect to the appropriate behavioral outcome measures. Transgenic manipulations did not yield conclusive information, while blockade of peripheral BDNF and/or TrkB signaling can decrease pain-like behaviors, particularly in models of prolonged or chronic pain, although important differences in BDNF effects were reported in different pain models (Table 2).

## 4. Spinal Nociception

The pronociceptive role of BDNF in the spinal cord has been extensively studied at the molecular, cellular, and behavioral levels. In fact, most of the information about the contribution of BDNF to pain processing originates from research on spinal mechanisms.

### 4.1. Expression and Localization

There is some controversy about the cellular source of BDNF in the spinal cord. Despite the evidence for microglia as a main source of BDNF in the spinal cord [30], immunostaining analyses showed that only a small subset of cells immunoreactive for the microglia marker Iba-1 co-expressed BDNF in dorsal horn laminae I-II of naïve postnatal [144] or of chronic post-ischemic pain (CPIP) [134] rats. Additionally, in double transgenic *BDNF-Cre/floxed-tdTomato* mice, BDNF seemed to be selectively expressed by neurons and not by glial cells in lamina I-V of the spinal cord [37]. The colocalization of GFP-expressing microglia with BDNF immunostaining in the spinal dorsal horns of transgenic CX3CR1^+/GFP^ mice after partial nerve ligation (PNL) [130] was not confirmed by subsequent studies. On the other hand, in primary microglia cultures, morphine-induced BDNF release was linked to ATP-mediated P2X4R activation [129,170], which was mediated by SNARE (soluble N-ethylmaleimide-sensitive factor attachment protein receptor) and p38/MAPK (mitogen-activated protein kinase) mechanisms [129]. Increased BDNF has additionally been shown to contribute to increased spinal microglia activation in neuropathic (streptozotocin-induced neuropathic pain and SNI) [171,172] and inflammatory (experimental autoimmune prostatitis) [173] pain models, and higher BDNF levels in microglia of the spinal nucleus of the tri-geminal nerve were associated with increased trigeminal allodynia in male rats [174].

Increased BDNF and TrkB levels were found in different pain conditions. In a model of inflammatory pain induced by systemic administration of NGF, BDNF immunoreactivity in the superficial layers of spinal dorsal horns was significantly enhanced compared to control in neonatal rats [154]. BDNF immunoreactivity in the ipsilateral (to the injury) superficial layers of the spinal cord was significantly enhanced after pSNL surgery compared to the contralateral side [175]. Similarly, an upregulation of BDNF mRNA and protein levels was detected in the superficial layers of spinal cord in rats with bilateral cervical facet joint distraction compared to a sham control group [156], while enhanced BDNF protein expression was found in the spinal L4–L5 segments ipsilaterally to the side of mammary gland carcinoma cells implantation in a model of bone cancer pain, and this upregulation was mediated by the activation of the proteinase-activated receptors 2 (PAR2)/-NFκB pathway [176].

Several studies focused on the presence of the TrkB receptor and its functional full length (TrkB-FL) isoform as potential targets and mediators of BDNF signaling. Electron microscopy analyses showed that TrkB-FL receptors were expressed postsynaptically on the dendrites and somata of second order neurons in the mouse and rat spinal dorsal horns [144,149], which led to the concept that BDNF released from afferent nerve terminals could act on pre- and postsynaptic elements to modulate nociceptive messages. However, only a small portion of TrkB-FL immunoreactive dendrites was found to form synapses with BDNF immunoreactive axons, though this was explained with technical limitations [149]. Additionally, enhanced BDNF and TrkB mRNA and protein levels were detected in the spinal cord of diabetic rats (STZ model) [157]; they were also increased in rats after C-fiber stimulation and in the CFA model [155] and in mice with neuropathic pain in the experimental autoimmune encephalomyelitis (EAE) model of multiple sclerosis [177]. Phosphorylation of TrkB is a critical mechanism for BDNF actions. The decrease in spinal phosphorylated TrkB protein levels by the blockade of TrkB autophosphorylation with an antagonist (1NM-PP1, a small protein derivate of the protein kinase inhibitor protein phosphatase 1) in *TrkB^F616A^* knock-in mice blocked the mechanical hypersensitivity in the capsaicin model of inflammatory pain [178].

The spinal cord seems to be a key structure for BDNF actions. There is good evidence for the presence of BDNF in spinal neurons. Still, it is commonly accepted that BDNF is released from microglia to act postsynaptically on neurons in the spinal cord (see Section 4.2), although the actual evidence for microglia-derived BDNF is not quite strong, with opposite findings (see Section 2.4.1), and therefore remains a knowledge gap.

### 4.2. Cellular Functions

BDNF signaling in the spinal cord plays a role in pain-related central sensitization and the regulation of neurotransmitters and neuromodulators.

In spinal cord slices from normal rats, BDNF application depolarized lamina I dorsal horn neurons and converted GABA-A receptor-mediated hyperpolarizing responses into depolarizing responses in a third of the recorded cells [30]. Importantly, in slices obtained from rats with peripheral nerve injury (PNI), treatment with a function-blocking antibody against the TrkB receptor (anti-TrkB) restored the hyperpolarizing GABA-induced effects that were lost in the untreated group, suggesting that endogenous BDNF is required for the pain-induced changes; here, BDNF was shown to be released from microglia and to act via TrkB [30].

BDNF application also significantly increased the responses of spinal neurons induced by NMDA or C-fiber stimulation in spinal cord slices, while a facilitatory trend was observed for A-fiber stimulation [154,179]. These effects were reversed by pretreatment with a BDNF-sequestering antibody (TrkB-IgG) [154]. Moreover, BDNF induced Ca^2+^ oscillations in lamina II neurons and resulted in an increase in the frequency, but not amplitude or decay time, of miniature excitatory synaptic currents (EPSCs) in dorsal horn neurons, suggesting the involvement of presynaptic mechanisms [144]. These effects were blocked by the application of a TrkB antagonist (K252a) or anti-trkB antibody (IgG1—clone 47), by the co-administration of AMPA and NMDA receptor antagonists (NBQX and D-AP5, respectively), and by a substance P-NK1 receptor blocker (L-732-138), suggesting that BDNF has facilitatory effects on glutamatergic and peptidergic transmission through TrkB activation [144]. Pretreatment with BDNF resulted in the failure of acutely applied capsaicin to induce Ca^2+^ oscillation in lamina II neurons, which could be explained by occlusion, consistent with BDNF engaging the release of neurotransmitters from nociceptive terminals [144]. In spinal cord slices from rats treated systemically with NGF to induce an inflammatory pain state, TrkB-IgG superfusion decreased the enhanced responses of spinal neurons to C-, but not A-, fiber stimulation, suggesting that BDNF signaling mediates spinal nociceptive processing in inflammatory pain [154]. Likewise, the i.th. injection of the TrkB-Fcchimera (to sequester BDNF) decreased excitatory synaptic responses (EPSCs) of lamina II neurons to the electrical DRG stimulation in a bone cancer-induced rat pain model [176]. Importantly, BDNF-mediated facilitatory effects were associated with the activation of the PLC/PKC pathway [175,179] that is linked to TrkB signaling (see Section 2.2).

It should be noted that early evidence from preclinical studies pointed to antinociceptive properties of exogenous BDNF at the spinal level under normal conditions, mainly mediated by an increased release of GABA from spinal interneurons. In an isolated dorsal horn preparation, BDNF bath application decreased the electrically or capsaicin-induced release of substance P from sensory neurons through concerted mechanisms involving GABA-B and TrkB signaling [180]. Importantly, in the same experimental setup, naloxone application failed to block BDNF-related effects, suggesting that the opioid system was not required in this process. Additionally, exogenous BDNF facilitated the release of GABA caused by K^+^ depolarization through TrkB receptors under normal conditions [180] and restored GABA levels that were depleted in a neuropathic pain model (7 days after SNL surgery) [181].

### 4.3. Behavioral Studies

A large body of evidence supports the involvement of BDNF in the development of pain at the level of the spinal cord (Table 2). A single i.th. injection of BDNF or extended delivery using a BDNF-transducing recombinant adenovirus (adBDNF) caused tactile allodynia and thermal hyperalgesia in naïve animals [30,175,182], and these effects were reversed by the pretreatment with antisense oligonucleotide against TrkB-FL mRNA that downregulated the expression of the receptor [182]. Heat hyperalgesia was also produced in normal rats by the i.th. injection of a high-affinity TrkB ligand, neuroptrophin-4/5 (NT-4/5), providing further evidence for TrkB-mediated behavioral effects [182]. Disruption of BDNF-TrkB signaling by the spinal application of antisense oligonucleotide against BDNF mRNA or anti-BDNF antibody or a BDNF-sequestering fusion protein (TrkB–Fc) reversed the enhanced mechanical and thermal responses in neuropathic (PNI, pSNL, or SNL) [30,153,175,183], bone cancer [176], bilateral cervical facet joint distraction [156], and carrageenan-induced inflammatory [182] pain models. In adult rats, i.th. injection of TrkB-IgG ameliorated nociceptive behaviors in both phases of the formalin test when animals were previously primed with systemic NGF injection compared to a saline pretreated control group and improved thermal withdrawal responses in a carrageenan-induced inflammatory pain model [154], suggesting that endogenous BDNF is involved in inflammatory pain.

There is evidence for the release of BDNF from microglial cells as a mechanism of pain at the spinal level. ATP-challenged microglia have been shown to cause tactile allodynia that was associated with the release of BDNF, demonstrating for the first time the association between BDNF and microglia in the modulation of pain behavior [30]. In fact, ATP-stimulated microglia previously treated with anti-TrkB or TrkB–Fc or transfected with BDNF siRNA failed to evoke changes in the mechanical withdrawal thresholds when delivered in the spinal cord of rats [30]. In support of these findings, P2X4 receptor-mediated release of BDNF by microglia activation with ATP was shown [129], and transgenic mice lacking P2X4 receptors (*P2X4^−/−^*) did not develop mechanical allodynia in PNL and SNI models of neuropathic pain via impaired BDNF release mechanisms in the spinal dorsal horns [130]. In the SNI model, the neuronal KCC2 downregulation induced by microglial BDNF release in the spinal dorsal horn led to dynamic mechanical allodynia; inhibiting microglia (and subsequent BDNF secretion) suppressed the induction of mechanical allodynia in male mice [184]. Conditional knockout of BDNF from microglia also prevented pain hypersensitivity in male mice with peripheral nerve injury [108]. BDNF-related crosstalk between glial cells has also been reported to influence pain behavior. Exogenous activation of spinal microglia with a CSF1 receptor agonist increased BDNF secretion and promoted mechanical allodynia in naïve rats, whereas inhibition of astrocytic-microglial CSF1-CSF1 receptor signaling with PLX-3397 (a CSF1 receptor antagonist) prevented BDNF release and relieved mechanical allodynia and thermal hyperalgesia in rats with ischemic pain [134]. Inhibiting spinal astrocyte activation with the BDNF/TrkB inhibitor ANA12 alleviated mechanical allodynia in a mouse partial crush injury model [185]. Together, the data suggest a pro-nociceptive role of BDNF within the spinal cord, and interventions that modulate BDNF signaling by targeting microglia and astrocytes show promise as potential therapeutic strategies for neuropathic pain conditions. It should be noted, however, that the colocalization of BDNF and microglial (e.g., Iba-1) markers in immunostaining has never been shown (see Section 4.1). This seems to be a controversial matter, perhaps due to technical limitations. Advanced technologies, such as single-cell transcriptomic analyses, offer the opportunity to resolve this important knowledge gap.

Importantly, a sexual dimorphism of BDNF signaling in microglia was reported with respect to pain processing. I.th. administration of an NGF/BDNF inhibitor (Y1036) or the BDNF-sequestering fusion protein TrkB-Fc and tamoxifen-induced Cre-loxP-mediated deletion of the Bdnf gene in CX3CR1-positive cells blocked SNI-induced mechanical allodynia in male, but not female, mice [186].

It should be noted that early reports point to antinociceptive mechanisms of spinal BDNF (see the last paragraph in Section 4.2). In these studies, i.th. injection of BDNF ameliorated thermal, but not mechanical, withdrawal thresholds in the injured paws of SNL rats (7 days after surgery) without affecting the responses of the contralateral paw, and this effect was blocked by a GABA-B receptor antagonist (CGP55445), suggesting that BDNF effects involved GABA-B signaling [181]. Surprisingly, similar effects were observed in normal conditions [180].

## 5. Pain Processing and Modulation in the Brain

### 5.1. Expression and Localization

BDNF signaling has been investigated at different supraspinal levels in the brainstem and brain in several models of pain.

In the brainstem, enhanced BDNF protein and immunomarker levels were found in the periaqueductal gray (PAG) in an inflammatory (CFA) pain condition [187]. Moreover, upregulation of TrkB (full-length but not truncated) and TrkB phosphorylation were detected in the rostral ventromedial medulla (RVM) of CFA rats [187], suggesting that the BDNF neurons projecting from PAG to RVM are activated in the inflammatory pain model. The PAG-RVM system is a critical component of the descending pain modulatory system [188]. Importantly, TrkB was shown to be expressed in the RVM serotoninergic neurons that project to the spinal cord as part of the descending pain control pathway [189]. In vitro studies performed in RVM slices revealed that the facilitatory effects of BDNF were associated with the tyrosine phosphorylation of the NMDA NR2A subunit through the IP3, PKC, and Src signaling pathway [187]. Higher BDNF protein levels in the RVM were detected in a model of pain induced by the combination of plantar incision with presurgical (24 h) paradoxical sleep deprivation as compared to the injury group [190], suggesting the critical involvement of BDNF signaling in nociceptive responses aggravated by sleep impairments. In the VTA, increased BDNF protein level and release were observed in CCI mice.

Within the limbic system, increased BDNF protein levels were detected in the central nucleus of amygdala (CeA) of CFA animals, which were associated with reduced activity of the transcriptional repressor histone dimethyltransferase G9a, supporting the idea of BDNF facilitatory effects in the amygdala [191]. Similarly, BDNF mRNA and protein expression was upregulated in the medial thalamus (MT) of central poststroke pain (CPSP) rats [192]. In a pain condition induced by chronic intermittent stress (CIS) followed by the induction of thermal injury (burn), increased BDNF, TrkB, and phosphorylated TrkB protein levels were observed in the hypothalamus, but not mPFC, as compared to the un-stressed group [193]. On the other hand, in the thalamic paraventricular nucleus, pro-BDNF and BDNF were downregulated in chronic restraint stress (CRS) mice [194]. In the reward system, BDNF protein and release were found to be upregulated NAc of neuropathic (CCI) mice [195].

In the cortex, BDNF mRNA and protein levels were upregulated in the ACC of rats in models of bone cancer [196], inflammatory (CFA) [109] and neuropathic (SNI) [197,198] pain. Importantly, BDNF immunostaining was increased in the contralateral (to the injury) S1 hindlimb portion of rats with CFA-induced inflammatory pain and was detected in GFAP-, Iba-1-, and NeuN-expressing cells, suggesting that BDNF is expressed in astrocytes, microglia, and neurons [109]. Accordingly, fluorescence in situ hybridization (FISH) experiments detected a higher percentage of BDNF mRNA and number of BDNF mRNA puncta in microglia in the contralateral (to the injury) S1 of neuropathic mice (SNI model) as compared to a sham group [108]. Interestingly, BDNF did not show changes at the protein or mRNA levels in PFC of SNI rats [197], and decreased BDNF levels were found in the IL but not prelimbic (PL) cortex of CFA rats [199], suggesting region-specific changes in BDNF in the mPFC. These bidirectional changes in cortical BDNF expression in pain conditions were also detected at the receptor level. TrkB mRNA and protein expression was enhanced in the ACC, but not PFC, in neuropathic (SNI) pain [197]. Reduced levels of BDNF were also observed in the hippocampal dentate gyrus (DG) [200] and CA1, as well as in the IL cortex [201], in an inflammatory (CFA) pain condition. Similarly, BDNF was found to be downregulated, while TrkB was upregulated, in hippocampal tissue in a mouse model of thalamic hemorrhage-induced CPSP [202].

In contrast to the periphery and spinal cord, supraspinal changes in BDNF in pain seem to be dependent on the targeted (sub-)region. Overall, a shift towards increased BDNF expression is observed in the brainstem and limbic regions in pain conditions. Within the cortex, the picture is less clear, and mixed and regional differences in pain-related expression change have been detected.

### 5.2. Cellular Functions

The cellular effects of BDNF signaling in supraspinal structures related to pain are not well understood but have become the focus of recent studies. Despite differential changes in the level of BDNF in different brain and brainstem structures (see Section 5.1), BDNF has been shown to primarily increase neuronal activity in these areas in pain models. For example, pain-induced upregulation of BDNF was associated with increased neuronal activity or evoked synaptic responses in the same areas such as S1 (SNI) [108], MT (CPSP) [192], nucleus raphe magnus (NRM) (CFA) [203,204], and trigeminal nucleus caudalis (TNC) (trigeminal allodynia induced by inflammatory soup) [174]. Importantly, these pain-induced abnormal neuronal activities were decreased by the application of TrkB antagonists, TrkB-IgG fusion protein, a BDNF scavenger, or depletion of BDNF [108,174,192,203,204]. On the other hand, inflammatory (CFA) pain-induced downregulation of BDNF in the ventral hippocampal CA1 to infralimbic cortex (vCA1-IL) pathway was accompanied by lower spontaneous neuronal firing and gamma power in the IL, decreased vCA1 to IL information flow, weakened phase-amplitude coupling (PAC) between vCA1theta phase and IL gamma amplitudes in in vivo electrophysiological experiments [199,201]. Infusion or overexpression of BDNF increased neuronal activity in the IL and normalized disrupted vCA1-IL connectivity in the pain model [199,201]. Similar to the beneficial effects of BDNF-induced neuronal activations in the vCA1-IL pathway under pain conditions, morphine-induced analgesia was mediated by BDNF-TrkB signaling and increased neuronal activity (c-Fos) in the amygdala (CeA) and BNST, which was decreased by the deletion of BDNF in the PB [205].

Several mechanisms have been linked to BDNF signaling in the brain. Upregulation of BDNF in inflammatory (CFA) and chronic (PNI or CPSP) pain conditions resulted in a TrkB-mediated downregulation or increased phosphorylation of KCC2 to disrupt Cl^−^ homeostasis and increase neuronal excitability [192,204,206]. Furthermore, multiple lines of evidence suggest that BDNF-TrkB signaling engages glutamatergic neurotransmission in pain conditions, including the upregulation of NMDA receptor 2B subunit (NR2B) [196,198], phosphorylation of AMPA receptor GluA1 subunit [203], and excitatory amino acid transporter (EAAT3) [174], to mediate increased excitatory synaptic transmissions and excitability or activation in pain. Downregulation of BDNF in a chronic neuropathic pain (CCI) model impaired the maintenance, but not induction, of hippocampal LTP, which was ameliorated by increasing BDNF in the hippocampus [207]. In the NRM, the exogenous application of BDNF increased frequency of mIPSCs and the accumulation of GAD65 in synaptic terminals under normal conditions [208], suggesting that BDNF-TrkB signaling can also modulate the release of inhibitory neurotransmitters. The effects of exogenous BDNF were lost in the CFA pain model due to increased endogenous BDNF [208]. Additionally, recent studies showed TrkB-dependent phosphorylation of ERK and CREB in inflammatory (CFA) and bone cancer pain models [109,196]. Activation of ERK-CREB signaling by BDNF could modulate gene expression and lead to long-lasting structural neuroplasticity underlying chronic pain conditions [209]. In line with this notion, systemic depletion of BDNF decreased neuropathic pain-induced dendritic spine remodeling in S1 [108], whereas rescue strategies to upregulate BDNF in the hippocampus (CA1) mitigated the reduction in dendritic spine and PSD-95 in neuropathic (CCI) pain [207] and promoted neurogenesis in an inflammatory (CFA) model of pain [200]. These findings collectively suggest that region-specific bidirectional changes in BDNF-TrkB signaling in supraspinal structures in pain conditions correlate with neuronal activity changes and neuroplasticity that can be mitigated by normalizing BDNF-TrkB signaling (Table 1).

### 5.3. Behavioral Studies

Pro- and anti-nociceptive effects have been reported in different brainstem regions. In the RVM, the injection of BDNF in naïve animals induced thermal hyperalgesia and mechanical allodynia [187,189] through NMDA receptors [187]. Conversely, suppression of BDNF-TrkB signaling in the RVM attenuated thermal hyperalgesia in an inflammatory pain model (CFA) [187] and paradoxical sleep deprivation-induced mechanical hypersensitivity in an incision pain model [190]. Interestingly, the injection of BDNF into the RVM in serotonin (5-HT)-depleted animals was antinociceptive, rather than pronociceptive [189], suggesting an important role of the 5-HT system in the pronociceptive effect of BDNF signaling in the RVM. The concentration of BDNF appears to be a key factor in its bidirectional effects on descending facilitation versus inhibition, because higher doses of exogenous BDNF in the RVM were antinociceptive, whereas lower doses were pronociceptive in naïve animals [187]. Consistent with the antinociceptive role of BDNF signaling in the RVM, blockade of TrkB in the RVM reversed histone deacetylase (HDAC) inhibitor-induced analgesic effects in an inflammatory pain model (CFA) [208]. BDNF signaling in the VTA may have beneficial effects in pain conditions, because overexpression of BDNF improved spatial memory formation in a neuropathic pain model (CCI), while BDNF knockdown blocked spatial memory improvement induced by the chemogenetic activation of DG-projecting VTA dopaminergic neurons [210].

In other supraspinal structures, the role of BDNF in pain modulation remains unknown or controversial. For example, infusion of BDNF into the midbrain near the (PAG) and dorsal raphe nuclei induced antinociceptive effects in naïve and formalin-injected animals [211,212,213]. In contrast, injection of BDNF into the PAG reversed the analgesic effect of transcranial direct current stimulation (tDCS) in a knee osteoarthritis model [214], pointing to a pronociceptive effect of BDNF in the PAG. In the central nucleus of the amygdala (CeA), the infusion of BDNF in naïve animals induced thermal hyperalgesia, promoted morphine reward, and rescued impaired morphine-induced CPP in animals with knockdown of a transcriptional regulator methyl CpG-binding protein 2 (MeCP2) [191]. Conversely, blockade of BDNF signaling in the CeA inhibited thermal hyperalgesia and morphine-induced CPP in an inflammatory pain model (CFA) [191]. On the other hand, intra-CeA injection of a BDNF scavenger (TrkB-Fc) decreased morphine-induced analgesia in naïve animals [205], suggesting perhaps an antinociceptive, rather than pronociceptive, role of endogenous BDNF signaling in the CeA BDNF. A potential source of BDNF in the CeA may be the parabrachial (PB) input because localized deletion of BDNF in the PB also decreased morphine-induced analgesia without affecting basal nociceptive responses and anxiety-like behaviors [205], indicating an important contribution of BDNF signaling in the PB-CeA pathway to opiate analgesia. In the thalamus, overexpression of BDNF in the parafascicular nucleus alleviated anxiety- and depression-like behaviors as well as hyperalgesia, while BDNF knockdown induced the opposite results in a chronic restraint stress (CRS) model [194]. In contrast, a BDNF scavenger (TrkB-Fc) or a TrkB antagonist (CTX-B) into the MT reversed hypersensitivity, but not thermal allodynia, in a CPSP model [192], indicating subregion-specific differential roles of BDNF signaling in thalamic pain processing.

In subcortical regions, pronociceptive effects of BDNF signaling have been consistently reported in the NAc. Injections of BDNF into the NAc shell increased thermal hyperalgesia without modulating depression-like behaviors in the CUMS model [215], while intra-NAc injections of a BDNF scavenger (TrkB-Fc) and a TrkB antagonist (ANA-12) decreased thermal hyperalgesia in a neuropathic pain model (CCI) [195] and optogenetically induced hypersensitivity in naïve animals [216], respectively. Injections of BDNF into the NAc reversed the antinociceptive effects of pharmacological inhibition, achieved with the injection of a GABA-B receptor antagonist (baclofen) or a I_h_ blocker (DK-AH269), of ventral tegmental area (VTA) neurons in a neuropathic pain model (CCI) [195]. Conversely, thermal nociceptive responses induced by the intra-VTA injection of morphine were prevented by the injection of a BDNF scavenger into the NAc shell without affecting depression-like behaviors in the CUMS model [215]. Furthermore, selective knockdown of BDNF in NAc-projecting VTA neurons reversed thermal hyperalgesia in a neuropathic pain model (CCI) [195]. These findings suggest that the VTA-NAc pathway is an important site for the pronociceptive action of BDNF signaling in the NAc. In contrast to the VTA-NAc pathway, BDNF signaling in the VTA-mPFC pathway seems to contribute to depression-like, but not nociceptive, behaviors [215].

Region-specific pro- and anti-nociceptive effects of BDNF signaling have been reported in the cortex. BDNF injections or viral vector-mediated upregulation of BDNF in the ACC produced cold hypersensitivity [109] and conditioned place avoidance (CPA) [196,197], as well as clonidine-induced conditioned place preference (CPP) [198], in naïve animals. Furthermore, exogenous BDNF into the ACC reversed the spinal clonidine-induced pain relief in a neuropathic pain model (SNI) [198]. Conversely, intra-ACC injections of a TrkB antagonist (Tat-CTX-B) blocked pain-related behaviors in inflammatory (CFA) [109], neuropathic (SNI) [197,198], and bone cancer [196] pain models. Several lines of evidence suggest a critical involvement of NR2B and the ERK-CREB signaling pathway in these pronociceptive effects of BDNF signaling within the ACC [196,197,198]. It is important to note that these effects were not observed with intra-PFC injections of BDNF or the TrkB antagonist [196,197,198]. Interestingly, intra-ACC injections of anti-proBDNF, but not anti-BDNF, antibodies mitigated chronic unpredictable mild stress (CUMS)-induced anxiety- and depression-like behaviors [217], suggesting a specific contribution of proBDNF signaling in the ACC to these emotional–affective behaviors. Similar pronociceptive effects of BDNF have been reported in the S1 cortex [108,109] and appear to involve microglial BDNF based on local depletion of microglial BDNF in transgenic mice [108]. In contrast to these pronociceptive effects, BDNF infusion into the IL subregion of the mPFC alleviated thermal hyperalgesia and mechanical allodynia in an inflammatory pain model (CFA) [199] and failed to promote thermal hypersensitivity in CUMS mice [215], suggesting region-specific effects of BDNF and differential roles of mPFC subregions in pain modulation in line with previous work [218]. Inputs from the hippocampus may play a key role in this antinociceptive effect, since overexpression of BDNF in the vCA1-IL pathway alleviated spontaneous pain, thermal hyperalgesia, mechanical allodynia, and anxiety-like behaviors in an inflammatory pain model (CFA) [201]. Overexpression of BDNF in the dentate gyrus (DG) also produced analgesic and anxiolytic effects and attenuated cognitive impairment in an inflammatory pain model (CFA) [200], suggesting that the IL and hippocampus are important brain areas for BDNF-induced beneficial effects under pain conditions, which is consistent with the merging role of disrupted hippocampal–mPFC (PL) connectivity in pain hypersensitivity and cognitive deficits [219].

In summary, activation of BDNF/TrkB signaling by exogenous BDNF has mixed effects in the brainstem but pronociceptive properties in subcortical areas, such as NAc, under normal conditions. Within the mPFC, the picture is less clear, with BDNF in the ACC having facilitatory effects while inhibitory effects were observed when IL or hippocampal inputs to the IL were targeted (and also the hippocampus itself). Similarly, blockade of BDNF/TrkB signaling had antinociceptive properties in the brainstem and subcortical regions, but mixed effects were observed within the mPFC, pointing to potentially region-specific roles of BDNF signaling in different brain regions in pain modulation (Table 2).

## 6. Other Brain Disorders

The involvement of BDNF in the brain in pain states remains a relatively understudied domain and a clear picture has yet to emerge. However, BDNF-related signaling pathways in the brain play important roles in other neurological and psychiatric conditions, which may be relevant to pain conditions as they are frequently comorbid with a wide spectrum of disorders such as anxiety and depression [220,221,222]. Therefore, information about BDNF signaling in these disorders may help inform about its role in chronic pain and the neurobiological interplay between chronic pain and its neuropsychiatric components.

### 6.1. Depression

Abundant research has implicated BDNF in the pathophysiology of depression and in the mechanisms of action of antidepressants, and BDNF-related signaling has become a therapeutic target for depression disorders [223,224,225,226]. In the rat learned helplessness model of depression, infusion of BDNF into the midbrain [227] or bilaterally into the hippocampal dentate gyrus [228] produced antidepressive effects. In the latter study, blockade of BDNF-TrkB signaling with the broad spectrum Trk inhibitor K252a prevented these effects [228]. In mice, decreased levels of BDNF in the hippocampus and PFC were linked to depression-like behavior in an inflammation-induced mouse model of depression [229], and conditional knockout of BDNF in the forebrain attenuated the actions of the antidepressant desipramine [230]. Similarly, corticosterone-induced depression in mice was associated with hyperactive neuronal autophagy in the dentate gyrus, which triggered increased lysosomal degradation of BDNF in neurons; inhibition of this autophagy with selective short hairpin RNA (shRNA) reversed the decreased neuronal BDNF expression and led to increased antidepressive effects [231].

However, BDNF in the brain seems to have region-specific effects on depression-like states. In contrast to the decreased levels of BDNF in forebrain areas, increased BDNF in the ventral tegmental area (VTA)–NAc pathway has been linked to the onset of depression [232,233]. Infusions of BDNF into the VTA resulted in increased depression-like behavior, whereas local deletion of the gene encoding BDNF in VTA neurons (projecting to the NAc) had an antidepressant-like effect in mice in the social defeat stress model [234]. Increased levels of BDNF in the NAc were also associated with a depression-like phenotype in mice [229]. Together, the data suggest that while BDNF may exert antidepressive actions in forebrain regions such as the hippocampus and PFC, it may contribute to a depression-like phenotype in the mesolimbic pathway centered on the VTA and NAc [235]. Understanding the differential effects of BDNF in various brain regions with different roles in depressive states could further illuminate its complex functions in pain perception and modulation.

### 6.2. Schizophrenia

Clinical studies have consistently shown that schizophrenic patients have lower BDNF levels in the hippocampus [7], PFC [236], and parietal cortex [6] compared to normal individuals. At the preclinical level, rats with neonatal ibotenic acid lesions of the ventral hippocampus (a neurodevelopmental animal model of schizophrenia) showed decreased BDNF mRNA levels in the hippocampus and PFC [237,238]. Rats in the methylazoxymethanol acetate (MAM) model of schizophrenia had significantly decreased BDNF in the hippocampus compared to controls, which was associated with cognitive deficits in the acquisition and retention phases of the Morris water maze [239]. Mice with a forebrain-specific knockout of the TrkB receptor showed hyperlocomotion, cognitive impairments, and other stereotypical behaviors associated with animal models of schizophrenia [240]. More recently, BDNF-haploinsufficient mice with lower levels of BDNF in the amygdala, dorsal hippocampus, NAc, and PFC showed deficits in attentional set shifting, increased startle magnitudes, and prepulse inhibition deficits, which are behavioral phenotypes associated with schizophrenia models; interestingly, these endophenotypes were rescued by environmental enrichment [241]. Intracerebroventricular administration of BDNF decreased schizophrenic-like behaviors (startle response and disrupted prepulse inhibition) in the DBA/2J mouse strain, which presented several behavioral features relevant to schizophrenia [242]. The specific TrkB agonist 7,8-dihydroxyflavone promoted hippocampal synaptic plasticity and reversed cognitive deficits in a MK-801-induced rat model of schizophrenia [243]. Together, the data suggest that restoration of BDNF-mediated signaling in these brain regions could restore cognitive functioning, which is also an important dimension in the experience of pain.

### 6.3. Neurodegeneration

Neurodegenerative diseases encompass a wide range of neurological disorders, including Alzheimer’s disease, Parkinson’s disease, and Huntington’s disease. While there are no pharmacological treatments currently available to alter the pathophysiology or provide a cure, beneficial effects of BDNF on cognitive functioning have been established [244,245,246]. It has been proposed that the decreased level of BDNF in neurodegenerative diseases leads to dysregulated GABAergic transmission via altered GABA release and transport in astrocytes and neurons, as well as through a decreased transcription of the GABA-A receptor [246]. Pharmacologically (aminopropyl carbazole) induced increases in hippocampal BDNF levels ameliorated cognitive function in a mouse model of Alzheimer’s disease [247]. In animal models of Parkinson’s disease, BDNF administration or increasing BDNF levels through gene transduction via viral delivery have been shown to enhance the survival of dopaminergic neurons and protect dopaminergic transmission to the striatum (reviewed in [248]). Inactivation of BDNF in the mouse forebrain led to a Huntington’s disease-like behavioral phenotype [249], and inactivation of one BDNF allele in a Huntington’s disease mouse model led to an earlier and worse behavioral and motor phenotype with severe striatal neuron loss [250]. Overexpression of BDNF in the forebrain in a mouse model of Huntington’s disease improved the behavioral and motor phenotype and reduced neuropathological signs in the striatum [251,252]. Therefore, BDNF-mediated signaling within the brain may represent a promising therapeutic target to restore or protect cognitive function in pain and other conditions.

## 7. Conclusions

Although BDNF signaling in pain has been extensively studied throughout the pain neuroaxis (Figure 1), several knowledge gaps remain. In addition to the neuronal release of BDNF, accumulating evidence suggests a critical role of BDNF from the neuroimmune system as a pain mechanism, but despite that, the picture remains unclear. Peripherally, BDNF seems to be released by neuronal afferents into the spinal cord. Spinal BDNF promotes facilitation, but the cellular source of BDNF remains unclear. Several studies claimed that it is microglial BDNF acting on spinal neurons that serves pronociceptive functions, but this has not been demonstrated directly. Undoubtedly, neuronal BDNF plays a critical role in pain facilitation by acting on several cell types, including neuroimmune elements, and therefore engages neuron-to-glia interactions. Mixed and bidirectional effects of BDNF signaling were observed in the brainstem and brain in different models of pain, which may point to (sub-)region specific differences in BDNF function as well as subregion-specific pain mechanisms at the supraspinal level. Additionally, the cellular sources of BDNF in the brainstem and brain have not been fully identified and may contribute to the differential roles of BDNF in pain modulation. Finally, BDNF deficits and beneficial effects of BDNF have been reported in several neuropsychiatric diseases. The contribution of BDNF, its source(s), and signaling pathway(s) to pain mechanisms across the nervous system remain to be determined, especially with respect to neuroimmune signaling and sex differences.

**Table 1:** BDNF effects on cellular functions.

Intervention	Region and Assay	Species	Pain Model	Effect	Reference
**Periphery**					
BDNF	DRG culture (patch-clamp)	Rat	STZ-induced neuropathy	↓neuronal properties	[158]
	DRG culture	Rat	TNF-α treatment	↑substance P and CGRP release	[159]
*Avil-CreERT2* (condition BDNF knockout from primary sensory neurons)	Spinal WDR neurons (in vivo electrophysiology)	Mouse	Naïve	No effects	[165]
**Spinal cord**					
BDNF	Isolated hemisected spinal cord	Rat	Naïve	↑NMDA-induced, C- and A-fiber evoked responses	[154]
BDNF	Lamina II neurons in slice (patch-clamp)	Rat	Naïve	↑C-fiber evoked responses (EPSCs)	[179]
BDNF	Lamina II neurons in slice (Ca^2+^ imaging)	Rat	Naïve	↑Ca^2+^ oscillations	[144]
			Capsaicin challenge	↓Ca^2+^ oscillations	
	Lamina II neurons in slice (patch-clamp)		Naïve	↑EPSC frequency; No effects on EPSC decay or amplitude	
TrkB-IgG	Lamina II neurons in slice (patch-clamp)	Rat	NGF-induced inflammation	↓C-fiber evoked responses	[154]
TrkB-Fc chimera	Lamina II neurons in slice (patch-clamp)	Rat	Bone cancer-induced pain	↓DRG evoked EPSCs	[176]
BDNF	Isolated dorsal horn with dorsal root attached	Rat	Naïve	↓electrical- or capsaicin-induced substance P release	[180]
				↑K^+^-mediated GABA release	
			SNL	↑GABA release	[181]
BDNF	Lamina I neurons in slice (patch-clamp)	Rat	Naïve	↑GABA-mediated Ca^2+^ responses; depolarized E_anion_	[30]
anti-TrkB			PNI	hyperpolarized E_anion_	
**Brain and brainstem**					
TrkB-Fc	MT (in vivo electrophysiology)	Rat	CPSP	↓SNS-electrically evoked neuronal response	[192]
BDNF	NRM (patch-clamp)	Rat	Naïve	↑frequency and amplitude of AMPA mEPSCs	[203]
TrkB-IgG			CFA	↓AMPA EPSCs	
BDNF	NRM (patch-clamp)	Rat	Naïve	↓mIPSC frequency	[208]
				Depolarizing shift in EPSC and ↑excitability in MOR-expressing neurons	[204]
TrkB-IgG			CFA	Hyperpolarizing shift in EPSC and ↓excitability in MOR-expressing neurons	
BDNF				No effect on mIPSC frequency	[208]
pAAV2-hSyn-Cre-GFP, (AAV2-Retro) + pAAV2-CAG-DIO-BDNF-mCherry-3∗flag(vCA1-IL pathway-specific overexpression of BDN)	IL (in vivo electrophysiology)	Rat	CFA	↑spontaneous neuronal firing, power spectral density in low gamma band, gPDC	[201]
p156sinRRLpptCAG-BDNF (BDNF lentiviral vector)	S1 (hindlimb part)	Rat	CFA	↑LTP	[109]
*Cx3cr1^CreER/+^;Bdnf^fl/fl^* (systemic depletion of microglial BDNF)	Layer 5 S1 (in vivo two-photon imaging)	Mouse	SNI	↓spontaneous and mechanically induced Ca^2+^ activity	[108]
EE-induced BDNF increase	Hippocampus	Mouse	CCI	↑LTP maintenance (fEPSP)	[207]

CCI = chronic constriction injury; CGRP = calcitonin gene-related peptide; CFA = Complete Freund’s Adjuvant; CPSP = central poststroke pain; DRG = dorsal root ganglion; EE = environmental enrichment; fEPSP = field excitatory postsynaptic potentials; gPDC = generalized partial directed coherence; EPSCs = excitatory postsynaptic currents; IL = infralimbic cortex; LTP = long-term potentiation; mIPSC = miniature inhibitory postsynaptic currents; MT = medial thalamus; NGF = nerve growth factor; NRM = nucleus raphe magnus; PNI = peripheral nerve injury; S1 = somatosensory cortex; SM1 = somatosensory cortex; SNL = spinal nerve ligation; SNI = spared nerve injury; SNS = sciatic nerve stimulation; STZ = streptozotocin; WDR neurons = wide-dynamic-range neurons.

**Table 2:** BDNF effects on pain-like behaviors.

Intervention	Region/Assay		Species	Pain Model	Effect	Reference
**Periphery**						
Avil-CreERT2 (condition BDNF knockout from primary sensory neurons)	Nocifensive behaviors		Mouse	Formalin test	↓second phase	[37,165]
	Mechanical allodynia			SNI- or paclitaxel-induced neuropathy	No effects	[37]
	Mechanical and thermal hypersensitivity			CFA inflammatory pain		
	Mechanical allodynia			pSNL		[165]
				SNL	↓	
	Mechanical hypersensitivity			Hyperalgesic priming		
BDNF	Mechanical allodynia		Mouse	Normal	↑	[97]
				SNC	No effects	
	Thermal hypersensitivity		Rat	Normal	↑	[164]
	Weight-bearing deficits and mechanical allodynia		Rat	Normal	No effects	[168]
				MIA MNX	↓	
	Mechanical allodynia		Rat	Normal	↑	[160]
Anti-BDNF antibody				L5 spinal nerve lesion model	↓	
Ad-proBDNF (adenovirus vector-encoding proBDNF gene)	Mechanical allodynia		Mouse	Normal	↑	[167]
	Flitching and licking (second phase)			Formalin (0.5%)		
**Spinal cord**						
Anti- BDNF antibody	Thermal hyperalgesia		Rat	SNL	↓	[153]
BDNF						[181]
				Normal		[180]
	Mechanical allodynia and thermal hyperalgesia		Mouse		↑	[175] [182]
	Mechanical allodynia		Rat			[30]
adBDNF						
neuroptrophin-4/5	Thermal hyperalgesia		Mouse			[182]
Antisense oligonucleotide against BDNF mRNA				Carrageenan inflammatory pain	↓	
	Mechanical allodynia			pSNL		[183] [175]
TrK-Fc	Mechanical allodynia and thermal hyperalgesia					
			Rat	Bone cancer pain		[176]
				Bilateral cervical facet joint distraction		[156]
	Mechanical allodynia		Rat	PNI		[30]
ATP-challenged microglia with anti-TrkB, TrkB-Fc BDNF siRNA				Normal	No effect	
TrKB-IgG	Nocifensive behaviors		Rat	NGF primed in formalin test	↓	[154]
Y1036, TrkB-Fc, *Cx3cr1^CreER^ × loxP-Bdnf* (tamoxifen-induced Cre-loxP–mediated deletion of the Bdnf gene in CX3CR1-positive cells)	Mechanical allodynia		Mouse	SNL	↓	[186]
**Brain and brainstem**						
BDNF, p156sinRRLpptCAG-BDNF (BDNF lentiviral vector)	ACC	Cold hypersensitivity, CPA, clonidine-induced CPP	Rat	Naïve	↑	[109,196,197,198]
Tat-CTX-B				CFA, SNI, bone cancer pain	↓	
BDNF	IL	Thermal hyperalgesia and mechanical allodynia	Rat	Naïve	No effects	[199]
				CFA	↓	
pAAV2-hSyn-Cre-GFP, (AAV2-Retro) + pAAV2-CAG-DIO-BDNF-mCherry-3∗flag (vCA1-IL pathway-specific overexpression of BDNF)	vCA1-IL	Spontaneous nociceptive behaviors, thermal hyperalgesia, mechanical allodynia, anxiety-like behaviors	Rat		↓	[201]
pAAV-CMV-MCS-EGFP-3Flag (BDNF-specific overexpression)	ventral DG	Thermal hyperalgesia, mechanical allodynia, anxiety-like behaviors	Mouse			[200]
BDNF	NAc	Thermal hyperalgesia	Mouse	CUMS	↑	[215]
TrkB-Fc				Morphine-induced	↓	
				CCI		[195]
ANA-12				Optogenetically induced hypersensitivity		[216]
BDNF	CeA	Thermal hyperalgesia	Mouse	Naïve	↑	[191]
TrkB-IgG				CFA	↓	
TrkB-Fc	CeA	Morphine-induced analgesia	Mouse	Naïve	↓	[205]
oe-BDNF lentivirus (BDNF overexpression)	Parafascicular nucleus of thalamus	Anxiety-like behaviors and mechanical allodynia	Mouse	CRS	↓	[194]
sh-BDNF lentivirus (BDNF knockdown)					↑	
TrkB-Fc, CTX-B	MT	Mechanical allodynia	Rat	CPSP	↓	[192]
		Thermal hyperalgesia			No effects	
K252a	NRM	Mechanical allodynia	Rat	CFA	↓	[208]
BDNF	Midbrain (PAG-DRN)	Thermal hyperalgesia	Rat	Normal	↓	[211,212]
		Nociceptive responses		Formalin		[213]
BDNF	PAG	tDCS-induced analgesic effects	Rat	MIA	↓	[214]
AAV-eGFP-Cre virus	PB	Thermal hyperalgesia and mechanical allodynia	Floxed-BDNF mouse	Normal	No effects	[205]
BDNF	RVM	Thermal hyper-algesia and mechanical allodynia	Rat	Naïve	↑	[187,189]
		Thermal hyperalgesia		CFA	↓	[187]
				RVM 5-HT-depleted animals		[189]
rabbit anti-BDNF antibody	RVM	PSD-induced cumulative pain scores and mechanical allodynia	Rat	Incisional pain	↓	[190]

CFA = Complete Freund’s Adjuvant; CPA = conditioned placed avoidance; CPP = conditioned place preference; CPSP, central poststroke pain; CRS = chronic restraint stress; CUMS = chronic unpredictable mild stress; DG = dentate gyrus; DRN = dorsal raphe nucleus; MIA, monoiodoacetate; MNX = transection of the medial meniscal; MT = medial thalamus; NGF = nerve growth factor; PNI = peripheral nerve injury; NRM = nucleus raphe magnus; PAG = periaqueductal gray; PSD = paradoxical sleep deprivation; pSNL = partial sciatic nerve ligation; SNC = sciatic nerve crush; SNL = spinal nerve ligation; tDCS = transcranial direct current stimulation.

## Data Availability

Not applicable.
